# The Comparison of Soil Agrochemical and Biological Properties in the Multi-Cropping Farming Systems

**DOI:** 10.3390/plants11060774

**Published:** 2022-03-14

**Authors:** Aušra Rudinskienė, Aušra Marcinkevičienė, Rimantas Velička, Robertas Kosteckas, Zita Kriaučiūnienė, Rimantas Vaisvalavičius

**Affiliations:** 1 Department of Agroecosystems and Soil Sciences, Agriculture Academy, Vytautas Magnus University, K. Donelaičio Str. 58, LT-44248 Kaunas, Lithuania; ausra.rudinskiene@vdu.lt (A.R.); rimantas.velicka@vdu.lt (R.V.); zita.kriauciuniene@vdu.lt (Z.K.); rimantas.vaisvalavicius@vdu.lt (R.V.); 2 Department of Plant Biology and Food Science, Agriculture Academy, Vytautas Magnus University, K. Donelaičio Str. 58, LT-44248 Kaunas, Lithuania; robertas.kosteckas@vdu.lt

**Keywords:** *Carum carvi* L., enzymes, multi-cropping system, root biomass, soil properties

## Abstract

Multi-cropping systems play an important role in improving the quality of soil properties. A field experiment was carried at the Experimental Station of Vytautas Magnus University Agriculture Academy (Lithuania) in 2017 to 2019. The aim of the study was to compare agrophysical and biological properties of the soil in the multi-cropping systems of sole (spring barley, spring wheat, pea, caraway), binary (spring barley–caraway, spring wheat–caraway, pea–caraway) and trinary (spring barley–caraway–white clover, spring wheat–caraway–white clover, pea–caraway–white clover) crops. In the second and the third years of caraway cultivation, when solely caraway was grown, the total nitrogen content was significantly lower than in binary and trinary crops (8.5% and 17.4%, respectively). The results indicated that the highest organic carbon content was in the third year of caraway cultivation in trinary crop when caraway was grown with peas and white clover. In the third year, the highest saccharase and urease activity was found in trinary crop where caraway was grown with spring barley and white clover. A strong positive correlation was observed between the content of saccharase and urease and the total nitrogen, organic carbon, and potassium available in the soil. The results of the study suggest that multi-cropping is important for soil conservation and the sustainability of agro-ecosystems.

## 1. Introduction

Over the last five decades, advances in agriculture have made it possible to meet the world’s highest demands for food, feed, and fibre [1]. However, sustaining the needs of an ever-increasing population is challenging due to widespread urbanisation, severe land degradation, and climate change [2]. Successful adaptation to and mitigation of climate change through agricultural management requires the development of simple, cost-effective, and largely scalable approaches. Therefore, an agricultural management strategy is essential [3]. The solution to these challenges is to achieve the long-term sustainable use of resources, taking into account the ecological and economic aspects of sustainability. In essence, eco-efficiency is about achieving more agricultural production with fewer resources [4]. Multi-cropping systems could increase crop diversity and avoid vulnerability to biotic stress [5], as well as ensure the sustainability of agriculture and increase crop and food production while reducing land use [6,7].

Multi-cropping is the cultivation of two or more crops in the same field based on ecological principles [8,9,10]. The main differences between mono- and multi-cropping are that the cultivation of agricultural crops differs in the length of the growing season as well as in biological and agronomic characteristics [11,12]. According to Lizarazo et al. [12], multi-cropping reduces nutrient leaching into deeper layers of the soil, as well as the abundance of pathogens and weeds. Therefore, the stronger the ecosystem, the higher the possibility for the reduction in fertilisers and pesticides. In multi-cropping agroecosystems, a dense upper and lower root horizon protects the soil from water and wind erosion and improves the agrochemical, physical, and biological properties of the soil [13]. The arrangement of plant stems and leaves in multi-cropping may be in both vertical and horizontal directions [7]. The roots of agricultural plant species also have different arrangements [14], thus broadening the spatial distribution of roots. In the case of multi-cropping with pulses, the roots of the plants are intertwined, facilitating nitrogen supply both for legume crops and other plants growing together [15,16]. Such soils are rich in mycorrhizal fungi, which improve plant nutrition and growth [17], as well as activate soil enzymes [18]. In addition, the mycorrhizal fungal hyphal network significantly improves soil structure and, in particular, soil water retention properties, making mycorrhizal-associated agricultural plants more resistant to drought [19].

Plant roots are very important in all ecosystem processes: the formation of the carbon cycle, metabolism, the stability of soil and its structure, and soil organisms [20]. Therefore, different cropping system patterns could affect the enzymatic activity of rhizosphere soil and the carbon and nitrogen of soil microbial biomass, which affect soil carbon and nitrogen mineralisation [21]. On this basis, crops growing in the multi-cropping system form more abundant plant root biomass, which enables the uptake of more nutrients from the soil [22]. Well-developed roots cover a larger volume of soil, and as a result, plants absorb more elements than only phosphorus and potassium and also increase the content of organic matter [23].

One of the most important indicators of soil fertility and biological (enzymatic) activity is the activity of enzymes [24,25]. In addition, it is a viable way to assess soil quality [26]. Soil enzymes are produced by microorganisms and, to a lesser extent, by plants and animals. Their combined activity expresses the viability of the soil at a given time. Enzymes have a significant effect on the mineralisation of plant residues in the soil, the nutrient cycle, the organic matter accumulation, and the structure of the soil [18,27].

Soil enzymes are specific proteins that catalyse the various chemical processes in the cell, helping microorganisms to absorb insoluble substances. The most commonly studied enzymes, urease and saccharase, belong to the class of hydrolases. Hydrolases decompose chemical bonds between C–O, C–S, and C–N in the presence of water. Urease is responsible for nitrogen metabolism and saccharase—for organic carbon conversion processes in the soil [18]. Biota respiration intensity, nitrifying power, abundance of microorganisms, amounts of humus, available phosphorus and potassium, pH, and crop yield are closely dependent on soil enzymes [28]. It is also noted that changes in soil properties due to the application of different tillage systems are closely related with enzyme activity. Therefore, by applying sustainable farming systems, leaving more plant residues, we can stimulate the activity of these enzymes in the soil and increase the overall soil fertility [29,30].

Caraway (*Carum carvi* L.) is an important biennial plant of the celery (*Apiaceae*) family native to Europe, Asia, and North Africa [31]. Caraway is a commercial plant and herb that is used not only as a spice for food but also in the pharmaceutical industry [32]. As a biennial plant, it can be grown with such annual plants as peas, beans, as well as with various herbs such as mustard, dill, or coriander, and the seeds that ripen only in the second or the third year. Therefore, it is important to know whether multi-cropping system could be an effective agricultural practice to enhance or balance the nutrients cycling, alongside the higher yield of intercropped caraway. Moreover, studies of soil health focusing on enzyme activity, are important and scarce in Lithuania.

Consequently, this study was conducted to investigate the plant-soil nutrient contents in the multi-cropping system with and without nitrogen fertilizer application and explain the belowground mechanisms for the plant and soil nutrients content optimisation. In this study, we hypothesized that the application of the multi-cropping system would improve soil agrochemical and biological properties and protect soil from degradation and erosion in order to improve soil quality and also promote adaptation to climate change. The aim of the study was to determine and compare the agrochemical (total nitrogen, organic carbon, available phosphorus, available potassium) and biological (root biomass, saccharase, and urease activity) soil properties in sole, binary, and trinary crops.

## 2. Results

### 2.1. Total Nitrogen Content in the Soil

*First year of caraway vegetative season.* At the end of the growing season in 2017, the total nitrogen content was significantly higher in the soil under binary crops of spring barley with caraway and pea with caraway and a trinary crop of pea with caraway and white clover by 15.8, 17.4, and 19.3%, respectively, compared to the sole crops of the aforementioned plants (Table 1). The soil with the sole caraway crop was not significantly different from the binary and trinary crops in terms of total nitrogen content.

*Second year of caraway vegetative season.* After caraway harvesting in 2018, the total nitrogen content in the soil with caraway after spring barley (binary crop) and caraway with white clover after spring barley and spring wheat (trinary crop) was significantly higher—13.8, 12.1, and 13.0%, respectively—compared to the sole crops of the same plants (Table 1). The total nitrogen content in the soil with the sole caraway crop was found to be significantly (8.5 to 13.7%) lower than in binary and trinary crops.

*Third year of caraway vegetative season*. After caraway harvesting in 2019, the content of total nitrogen in the soil with caraway after spring barley and spring wheat with and without white clover was significantly higher (19.4, 14.5, 21.3, and 18.2%, respectively) compared to the soil with the sole crops (Table 1). The total nitrogen content in the bare fallow left after caraway harvesting was found to be significantly (9.2 to 17.4%) lower than in the soil, where caraway was grown with and without white clover.

### 2.2. Organic Carbon Content in the Soil

*First year of caraway vegetative season.* The organic carbon content in the soil under binary crops of spring barley with caraway, spring wheat with caraway, and pea with caraway and trinary crops of spring wheat with caraway and white clover and of pea with caraway and white clover was found to be significantly higher (by 16.3, 16.7, 9.0, 17.8, and 14.6%, respectively) than in the soil with the sole crops (Table 2). the organic carbon content of the soil with the sole caraway crop was found to be significantly higher than that of the binary crop of caraway with peas and the trinary crop of caraway with spring barley and white clover, 8.2 and 9.4%, respectively.

*Second year of caraway vegetative season.* The organic carbon content in the soils where caraway grew after spring barley with spring wheat (binary crop) and after spring wheat with white clover (trinary crop) was found to be significantly higher, by 16.0, 15.3, and 13.3%, respectively, than in the soils with sole crops (Table 2). the organic carbon content in the soil with sole caraway crop was not significantly different from that of the binary and trinary crops.

*Third year of caraway vegetative season.* After caraway harvesting in 2019, the organic carbon content in the soils where caraway grew after spring barley and spring wheat without and with white clover was found to be significantly, 12.9, 16.4, 13.6, and 15.7%, higher, by 12.9, 16.4, 13.6, and 15.7%, respectively, compared to sole crops (Table 2). The organic carbon content in the soil of bare fallow after caraway harvesting was found to be significantly lower, by 15.6 to 18.8%, respectively, than in the soil covered with binary and trinary crops.

### 2.3. Available Phosphorus Content in the Soil

*First year of caraway vegetative season.* At the end of the growing season in 2017, the available phosphorus content in the soil under binary crop of spring barley with caraway and trinary crop of spring barley with caraway and white clover was found to be significantly higher, by 34.9 and 27.1%, respectively, than those of the sole crops of the aforementioned plants (Table 3). The available phosphorus content in the soil with sole caraway crop was found to be significantly lower compared to its binary crop with spring barley and trinary crop with spring barley and white clover by 15.9 and 10.7%, respectively.

*Second year of caraway vegetative season*. After caraway harvesting, the available phosphorus content in the soil where caraway was grown after spring barley without white clover and together with white clover was found to be significantly higher, by 61.1 and 44.6%, respectively, than in the soil where spring barley was grown alone (Table 3). The available phosphorus content in the soil with sole caraway crop was not significantly different from that of the binary and trinary crops.

*Third year of caraway vegetative season*. After caraway harvesting in 2019, the available phosphorus content in the soil where caraway was grown after spring barley and spring wheat without white clover and with it was found to be significantly higher, by 33.1, 30.1, 19.7, and 24.8%, respectively, than in the soil under sole cropping (Table 3). The available phosphorus content in bare fallow soil after caraway harvesting was found to be significantly lower, by 8.5 to 23.7%, respectively, than in binary and trinary crops, except when caraway was grown after pea without and with white clover.

### 2.4. Available Potassium Content in the Soil

*First year of caraway vegetative season.* At the end of the growing season in 2017, the available potassium content in the soil with trinary crop of spring barley with caraway and white clover was significantly higher, by 14.9%, compared to the soil with a sole crop (Table 4). The soil with a sole caraway crop did not show any significant difference in the available potassium content compared to binary and trinary crops.

*Second year of caraway vegetative season.* After caraway harvesting, there was no significant difference in the available potassium content in the soil of sole, binary, and trinary crops (Table 4).

*Third year of caraway vegetative season*. The available potassium content in the soil where caraway grew after spring barley, spring wheat, and pea without white clover (binary crops) and with clover (trinary crops) was found to be significantly higher than in the soil of sole crops, 46.2, 32.9, and 30.2 and 62.8, 40.7, and 36.2%, respectively (Table 4). Available potassium content in the soil of bare fallow after caraway harvesting was not significantly different compared to binary and trinary crops.

### 2.5. Root Dry Biomass

**Figure 1:**
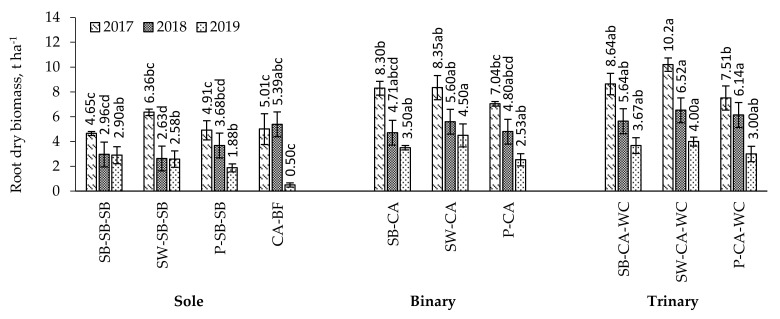
Root dry biomass in sole, binary, and trinary crops in 2017–2019. Note. CA—caraway; SB—spring barley; SW—spring wheat; P—pea; WC—white clover; BF—bare fallow. Differences between the averages of treatments marked with different letters (a, b, c, d) are significant (*p* < 0.05); error bars indicate the standard error.

*First year of caraway vegetative season.* When spring barley was grown in binary crop with caraway intercrop and when growing spring barley, spring wheat, and pea in trinary crop with caraway and white clover intercrop, significantly higher, by 78.5, 85.8, 60.4 and 53.0%, plant root mass was determined compared to sole crop of the aforementioned plants (Figure 1).

*Second year of caraway vegetative season.* In 2018, when caraway was grown after spring wheat harvesting without white clover (binary crop) and with white clover after spring barley, spring wheat, and peas (trinary crop), the plant root mass was significantly higher compared to that of sole crop—2.1, 1.9, 2.5, 1.7, and 2.5 times, respectively (Figure 1).

*Third year of caraway vegetative season.* In 2019, when caraway was grown after spring wheat harvesting (binary crop) and with white clover (trinary crop), the plant root mass was found to be significantly higher, by 74.4 and 55.0%, respectively, compared to sole crop (Figure 1). The plant root mass in bare fallow left after caraway harvesting was found to be significantly lower, by 5.1 to 9.0 times, respectively, compared to that of binary and trinary crops.

### 2.6. Saccharase Activity in the Soil

**Figure 2:**
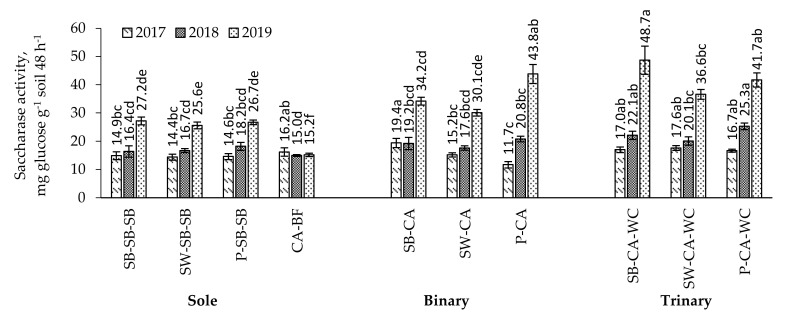
Saccharase activity in sole, binary, and trinary crops in 2017–2019. Note. CA—caraway; SB—spring barley; SW—spring wheat; P—pea; WC—white clover; BF—black fallow. Differences between the averages of treatments marked with different letters (a, b, c, d, e, f) are significant (*p* < 0.05); error bars indicate the standard error.

*First year of caraway vegetative season.* The highest saccharase activity was found in the soil of binary crop of spring barley with caraway (Figure 2). This could be influenced by the formation of a more abundant plant root system. Compared to the sole crop, except caraway, and binary crop of spring wheat and pea with caraway intercrop, the saccharase activity was found to be significantly higher, by 30.2, 34.7, 32.9%, 27.6, and 65.8%, respectively. The soil saccharase activity was found to be significantly higher, by 42.7%, in the trinary crop of pea with caraway and white clover compared to that in the binary crop of pea with caraway.

*Second year of caraway vegetative season.* The activity of enzyme saccharase in the soil in the second year of caraway growth was found to be higher than in the first year (Figure 2). This could be influenced by the higher content of organic matter in the soil. The activity of the enzyme saccharase in the soil where caraway was grown together with white clover after spring barley and pea was found to be significantly higher compared to sole crop—by 34.8 and 39.0%, respectively. The saccharase activity in the soil where caraway was grown with white clover after peas was found to be significantly higher, by 21.6%, than that in the soil where caraway was grown without clover.

The activity of enzyme saccharase was found to be significantly lower, from 34.0 to 68.7%, in the soil with a sole crop of caraway than in the soil where caraway was grown after pea (binary crop) and after spring barley, spring wheat, and peas in combination with white clover (trinary crop).

*Third year of caraway vegetative season*. The activity of enzyme saccharase in the soil in the third year of caraway cultivation was found to be higher than in the first and second years of cultivation (Figure 2). In the soil where caraway was grown after peas without white clover, and together with clover after spring barley, spring wheat, and peas, the saccharase activity was found to be significantly higher than in the soil where caraway was grown as the sole crop—64.0, 79.0, 43.0, and 56.2%, respectively. The saccharase activity in the soil where caraway was grown with white clover after barley was found to be significantly higher, by 42.4%, compared to the soil where it was grown without clover. The activity of enzyme saccharase in the soil with binary and trinary crops was found to be significantly higher, by 2.0 to 3.2 times, than in bare fallow left after caraway harvesting.

### 2.7. Urease Activity in the Soil

**Figure 3:**
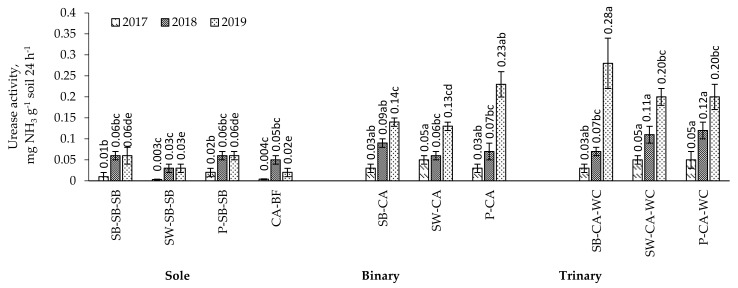
Urease activity in sole, binary, and trinary crops in 2017–2019. Note. CA—caraway, SB—spring barley; SW—spring wheat; P—pea; WC—white clover; BF—black fallow. Differences between the averages of treatments marked with different letters (a, b, c, d, e) are significant (*p* < 0.05); error bars indicate the standard error.

*First year of caraway vegetative season.* The activity of enzyme urease in the soil with binary crop of spring wheat with caraway and trinary crops of spring wheat and pea with caraway or white clover intercrops was found to be significantly higher compared to sole crop—16.7, 16.7 and 2.5 times, respectively (Figure 3). The activity of enzyme urease in the soil with sole crop of caraway was found to be significantly lower, by 7.5 to 12.5 times, than that of caraway grown in binary and trinary crops.

*Second year of caraway vegetative season.* The activity of enzyme urease in the soil in the second year of caraway cultivation was also found to be higher than in the first year (Figure 3). Urease activity in the soil with a trinary crop of caraway with white clover after spring wheat and pea was found to be significantly higher compared to sole crop by 3.7 and 2.0 times, respectively.

The activity of enzyme urease activity in the soil where caraway grew with white clover after spring wheat and pea was found to be significantly higher, by 83.3 and 71.4%, than that in the soil of binary crop. Urease activity in the soil with sole crop of caraway was significantly lower, by 2.2 to 2.4 times, than in the soil of the trinary crop after spring wheat or pea.

*Third year of caraway vegetative season.* The activity of enzyme urease in the soil was found to be significantly higher than in the first and second years of caraway cultivation (Figure 3). The highest urease activity was found in the soil of trinary crop of spring barley with caraway and white clover. The urease activity in the soil in which caraway was grown without white clover (binary crop) and together with it (trinary crop) after spring barley, spring wheat, and pea was found to be significantly higher compared to sole crop—2.3, 4.3, and 3.8 and 4.7, 6.7, and 3.3 times, respectively. The urease activity in the soil where caraway was grown with white clover after spring barley was found to be significantly higher, by 2.0 times, than that in the soil where caraway was grown without clover. The urease activity in bare fallow left after caraway harvesting was found to be significantly lower, by 6.5 to 14.0 times, compared to the soil where caraway was grown without white clover (binary crop) and in combination with white clover (trinary crop).

### 2.8. Principal Component Analysis (PCA)

**Figure 4:**
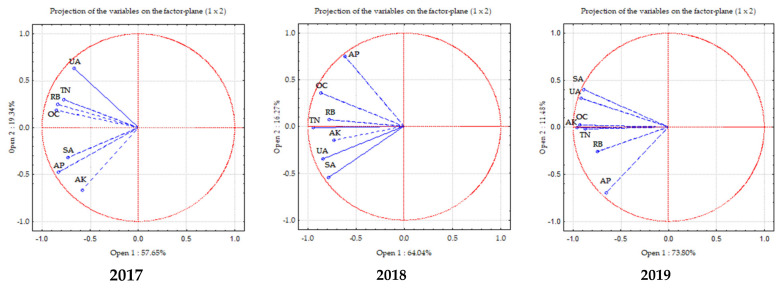
PCA of sole, binary, and trinary crops in 2017–2019. Note. UA—urease activity; TN—total nitrogen; RB—root dry biomass; OC—organic carbon; SA—saccharase activity; AP—available phosphorus; AK—available potassium.

PCA showed different correlations between individual parameters depending on the multi-cropping system used. Two groups of correlated parameters were identified in the first year (2017) of caraway cultivation (Figure 4). The first group of correlated parameters included total nitrogen, organic carbon, root biomass, and the activity of soil enzyme urease. The second group of correlated parameters included available phosphorus, available potassium, and the activity of soil enzyme saccharase.

In the second year (2018) of caraway cultivation, the activity of the soil enzyme urease was more dependent on the agrochemical soil properties (total nitrogen, available potassium, and organic carbon) and root biomass than on the activity of enzyme saccharase.

In the third year (2019) of caraway cultivation, the activity of the soil enzymes saccharase and urease correlated with the levels of total nitrogen, organic carbon, and available potassium.

## 3. Discussion

### 3.1. N, OC, P, K Contents in the Multi-Cropping System

Studies have shown that the soil nutrient balance was better maintained in soils with binary crops with caraway intercrop and trinary crops with caraway and white clover intercrop compared to sole crops. The total nitrogen, organic carbon, available phosphorus, and potassium in the soil increased the most in binary and trinary crops in the third year of caraway cultivation compared to the initial contents.

The results of other authors showed that the fixed nitrogen content in leguminous plants in multiple crops depended on several factors such as species, plant morphology, crop density, and total nitrogen in the soil [33]. The results of Andersen et al. [34] show that when forming multiple crops of legumes and other crops, the benefits of N_2_ fixation are partially lost if the selected plants have a stronger competing capacity than leguminous plants. Hauggaard-Nielsen et al. [35] stress that multi-cropping can have a positive impact on nutrient retention. Despite competition between plants, significantly higher total nitrogen levels were found in the soil of the second and third years of caraway cultivation compared to the sole crop (Table 1).

The soil organic carbon content increased significantly by 3.8–17.8% in 2017–2019 in the multi-cropping system (Table 2). The study confirmed that the availability of nutrients in the soil was significantly higher in binary and trinary crops compared to sole crops. Data from Adamu and Yusuf [36] showed the same trend of increasing organic carbon in multiple crops compared to sole crops. This can be explained by the fact that root exudates create favourable conditions for the nutrition of microorganisms and thus for plant nutrition. The soil is also positively affected by the multiple crop root system, which promotes the activity of micro-organisms. Studies have shown that carbon availability increased in the carbon cycle [37]. More nutrients important for plant mineral nutrition were released, while a denser soil cover reduced the rate of mineralisation and inhibited nutrient leaching.

The available phosphorus also depends on the organic matter content in the soil [38]. In the presence of available phosphorus deficiency and drought conditions, it has been observed that crops grown in mixtures absorb phosphorus released in the soil from leguminous plants directly, thus allowing for plant resilience [39]. In addition, the availability of organic phosphorus is also increased by a variety of leguminous crops. Muofhe, Dakora [40] found that growing legumes and cereals together increased the uptake of phosphorus due to the release of lactic anions that chelated Fe^3+^ and subsequently released phosphorus from FePO_4_. Studies have shown that leguminous plants released higher levels of phosphorus-mobilising exudates [41], which could also support phosphorus nutrition of other plant species in multiple crops with lower phosphorus uptake capacity [42]. Our experimental data confirmed that the phosphorus content in the soil was significantly increased with the cultivation of binary and trinary crops compared to sole crops (Table 3). This apparent effect may have been due to the higher accumulation of residues in the topsoil layer that were more resistant to decomposition. The intertwining of caraway roots with pea and white clover roots had a positive effect on the release of phosphorus from the soil, while at the same time, the remaining decomposing root parts contributed to the organic matter content in the soil.

In the third year of caraway cultivation, the available potassium was significantly higher in the soil with binary and trinary crops compared to sole crops (Table 4). The results of Nasar et al. [43] show that the cereal–legume multi-cropping system significantly increased the available potassium in the soil compared to the sole crop. The reason for this increase in available potassium is not fully understood; however, it may be due to the effect of cation antagonism between potassium and calcium when roots interact in multiple-crop conditions [37,44,45]. In addition, most of the potassium is accumulated in plant by-products (straw) [46]. Therefore, it can be argued that a thicker layer of plant residues in the third year of caraway cultivation may also affect the higher amount of potassium in the soil.

### 3.2. Root Biomass in the Multi-Cropping System

In the case of multi-cropping, resource uptake and competition between plant roots during the growing season is more evenly distributed, with peak nutrient uptake occurring at different stages. The results of the study showed that in the first year of caraway cultivation, the plant root mass was significantly higher in binary and trinary crops compared to sole crops—by 53.0 to 85.8%, respectively. A similar trend was observed in both the second and third years of caraway cultivation, with a significantly higher—from 1.7 to 2.5 times—plant root mass formed in binary and trinary crops compared to sole crops (Figure 4). Papa et al. [47] found that roots of different plant species took up moisture and nutrients at different rhythms and intensities. The interactions between plants and interspecific competition promote plant rooting [17]. Competition is also largely avoided during vegetation [43,48]. However, Bellostas et al. [49] received contradictory results: with a two-species multi-crop, the intertwining of plant roots in the early stages of growth led to negative competitive effects. According to the authors, two weeks after sowing the binary crop, plant dry matter production was reduced by 15–20% compared to the sole crop [49]. It can be argued that a more abundant and denser root system has a greater suction power, resulting in the better uptake of nutrients from the soil and supplying them to plants, making them better prepared for adverse environmental conditions.

### 3.3. Soil Enzyme Activity in the Multi-Cropping System

Changes in the saccharase activity in the soil depended mainly on the root mass and the amount of crop residues. The highest levels of enzyme saccharase were found in trinary crop in the third year of caraway cultivation (Figure 2). This means that multiple crops can stimulate the growth and development of plant roots, improving the biological and chemical properties of the soil. The highest saccharase activity was in the arable soil layer. It is mainly related to organic carbon, available phosphorus and potassium, CO_2_ release, and plant residues. Cui et al. [50] found that the activity of the soil enzyme saccharase in multiple crops was significantly correlated with the soil ammonium and nitrate nitrogen. This means that pH and total nitrogen were the main factors influencing soil enzyme activity [50]. The increase in saccharase activity indicates an acceleration of hydrolytic carbohydrate degradation and intensification of organic matter mineralisation processes in the soil [51]. This implies that multiple crops can improve soil nutrient cycle by improving the activity of soil saccharase enzymes.

The studies on urease activity showed that the reduction in the tillage intensity in the soil in the third year of caraway cultivation, where caraway was grown with white clover after barley (trinary crop), may have created a better biochemical soil environment, leading to a higher stable enzyme concentration (Figure 3). In fact, soil enzymes are responsible for nutrient cycles and availability [20,52]. In addition, the enzyme urease acts by hydrolysing the C–N bond of amides and releasing ammonia during nitrogen mineralisation [53]. Thus, higher activity of this enzyme in trinary combination of spring barley, caraway, and white clover suggests that crop residues had a positive effect on enzyme urease activity and subsequently improved the nitrogen availability and soil biological properties. Moreover, trinary crops combinations with a predominance of leguminous plants increased the levels of this enzyme in the third year of caraway cultivation. No mineral fertiliser was applied and the root system of the leguminous plants transformed the rhizosphere of the plants grown together. Biological nitrogen fixation by leguminous plants acidified the rhizosphere and released higher levels of soil enzymes (urease and other enzymes) as well [54]. Our data showed that the content of saccharase and urease was the most correlated with the total nitrogen, organic carbon, and available potassium in the soil (Figure 4).

## 4. Materials and Methods

### 4.1. General Experimental Conditions

**Figure 5:**
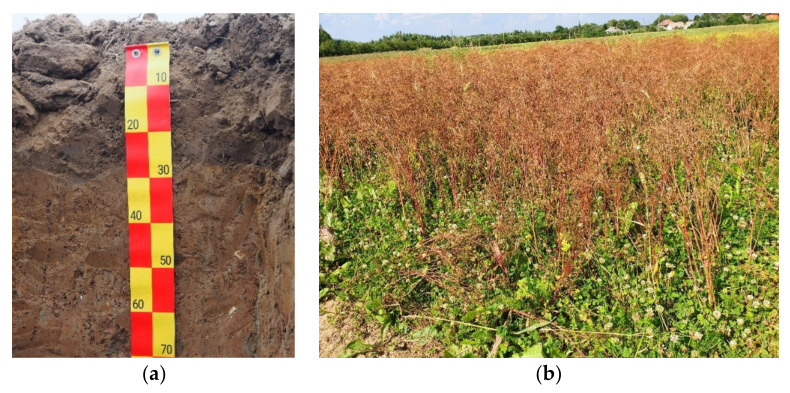
Experimental site: (**a**) actual, layers of the soil; (**b**) trinary crop of spring barley intercropped with caraway and white clover in the third year of caraway vegetative season in 2019.

A field experiment was carried out during three vegetative seasons (2017, 2018, 2019) in the Experimental Station of Vytautas Magnus University Agriculture Academy (VMU AA), Kaunas district, Lithuania (coordinates: 54°53′7.5″ N latitude and 23°50′18.11″ E longitude). The soil of the experimental site (Figure 5) was Endocalcaric Amphistagnic Luvisol, according to the World Reference Base (WRB) [55].

The texture of the topsoil is sandy loam, and the agrochemical properties are the following: pH_KCl_—6.70; organic carbon (OC)—0.91–1.08%; plant-available phosphorus (P_2_O_5_)—213–318 mg kg^−1^; and potassium (K_2_O)—103–125 mg kg^−1^. Thus, the above-listed soil characteristics show that the topsoil richness in organic carbon (OC) is low, but the available phosphorus (P_2_O_5_) corresponds to group V (very high content) and potassium (K_2_O) to group III (average concentration) according to the evaluation of agrochemical properties of Lithuanian soil [56].

**Figure 6:**
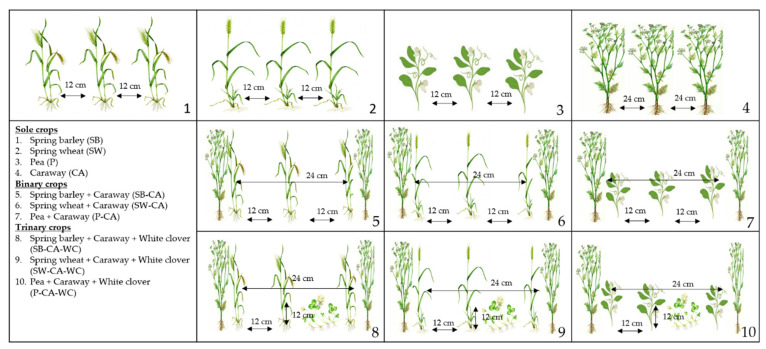
Experimental treatments: Four treatments were formed by sole crops, three treatments were combinations of caraway with the other crops as binary crops, and three treatments were trinary crops with white clover added to binary crops.

A 1-factor field experiment with 10 treatments was set up in 2017 (Figure 6). The experiment was performed in four replications, and a randomized complete block design (RCBD) was used. The size of each experimental plot was 60 m^2^ (5 m × 12 m), one replication block was 600 m^2^; 2 m buffer rows were left between the individual blocks. There were 40 plots in total in the experimental field.

The experimental field was ploughed in autumn 2016, and in spring 2017, it was cultivated with a germinator twice and fertilised with complex fertiliser NPK 8-20-30 (300 kg ha^−1^) applied with fertilizer spreader AMAZONE ZAM 1201 (Hasbergen, Germany). In spring of the first caraway vegetative season in 2017, sole spring barley (*Hordeum vulgare* L.) “Orphelia KWS” (160 kg ha^−1^), spring wheat (*Triticum aestivum* L.) “Quintus” (250 kg ha^−1^), pea (*Pisum sativum* L.) “Salamanca” (280 kg ha^−1^), and caraway (*Carum carvi* L.) “Gintaras” (7 kg ha^−1^) were sown at 12 cm interrow spacing. In binary and trinary crops, caraway was sown at 24 cm interrow spacing. In trinary crop, white clover (*Trifolium repens* L.) “Sūduviai” (2 kg ha^−1^) was sown into spring barley, spring wheat, and peas at 12 cm interrow spacing crosswise to the direction of other crops.

**Figure 7:**
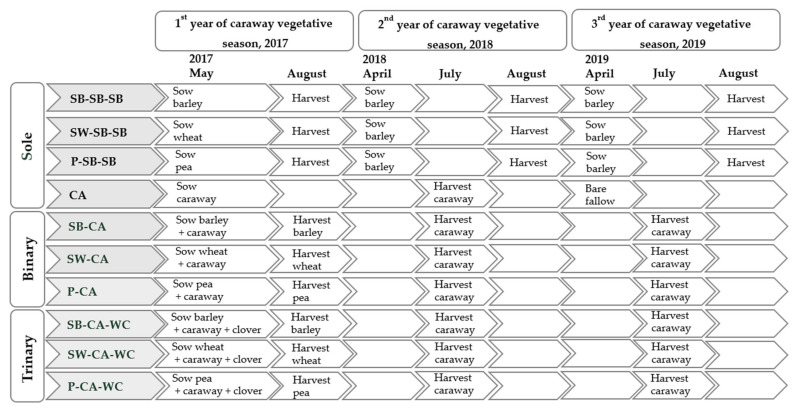
Timeline of the experiment. Note. CA—caraway; SB—spring barley; SW—spring wheat; P—pea; WC—white clover; BF—black fallow.

After the main crops—spring barley, spring wheat, and peas in 2017 and 2018—and after sole caraway crop in 2018, the harvested plots were disked and deeply ploughed. In the spring of 2018 and 2019, spring barley “Orphelia KWS” (180 kg ha^−1^) was sown (Figure 7).

In the first year of caraway vegetation (2017), sole spring barley and spring wheat crops and binary crops with undersown caraway were fertilised with ammonium nitrate at the rate of 180 kg ha^−1^, and trinary crops with undersown with caraway and white clover at the rate of 150 kg ha^−1^.

In the second (2018) and the third (2019) years of caraway vegetation, caraway was not fertilised, and no plant protection products were used.

Technology for the use of plant protection products in multi-cropping system (sole, binary, trinary crops) is presented in Table 5.

### 4.2. Meteorological Conditions

**Figure 8:**
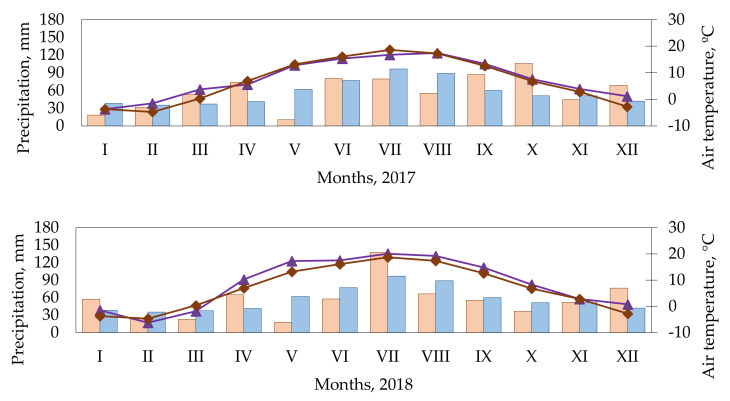

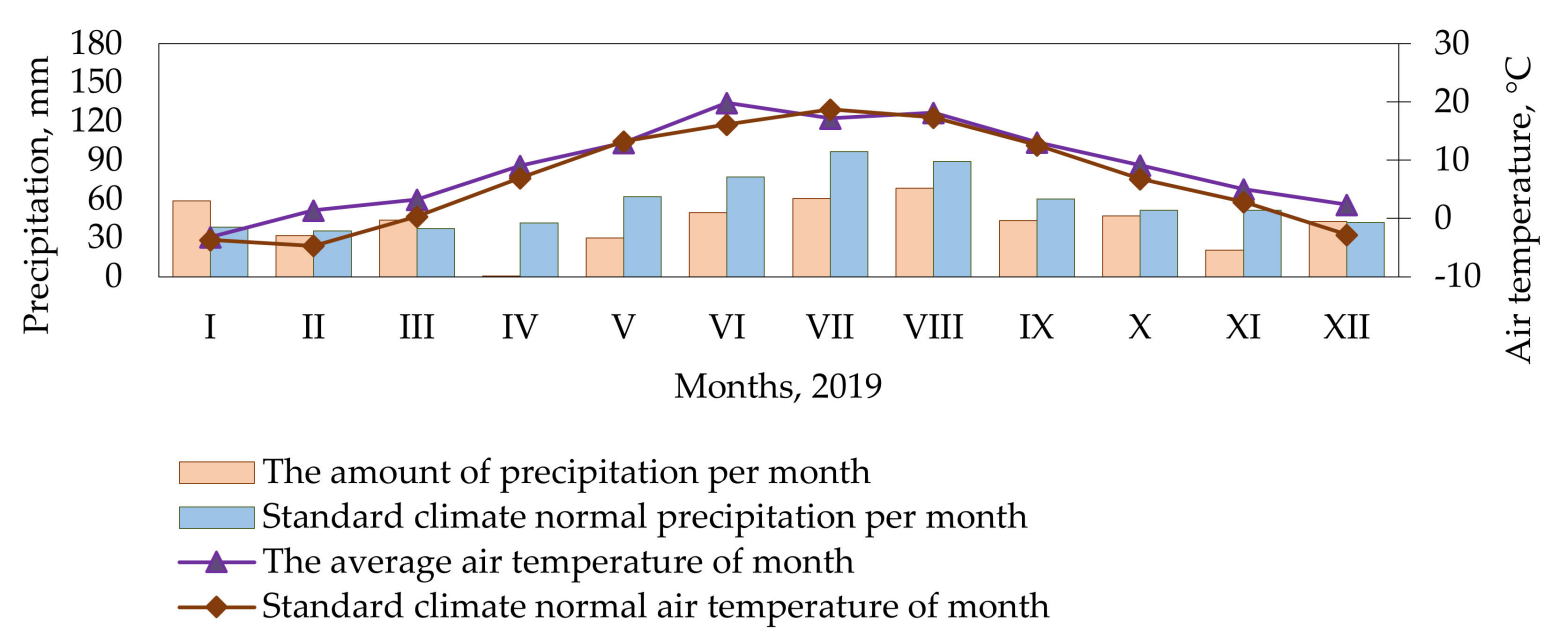
Meteorological conditions during the experimental period, Kaunas Meteorological Station, 2017–2019. Note. Long-term average is the data for 40 years (1974–2013).

April was cold and humid (Figure 8). May was very dry (hydrothermal coefficient (HTC) was 0.29). The amount of precipitation in June was close to the long-term average precipitation. July was cool with HTC of 1.53 (optimal humidity). In August, HTC was 1.00 (optimal humidity). September was warm with HTC 2.26 (excess humidity). In October, precipitation exceeded the long-term average of precipitation several times. In January 2018, the temperature was 2.2 °C higher than the long-term average. February and March were colder than usual. The temperature in April was 3.3 °C higher compared to the long-term average. The temperature in May was 4.0 °C higher than the long-term average, and the precipitation was 44.1 mm lower than usual. The monthly HTC was 0.33 (very dry), and the plants lacked moisture for growth. The temperature in June was 1.4 °C lower than the long-term average, and HTC was 1.10 (optimal humidity). The temperature in July was 1.4 °C higher than the long-term average, and the precipitation was 40.9 mm higher than usual, and HTC was 2.20 (excess humidity). August was warm with an HTC of 1.12 (optimal humidity). Temperatures were higher than usual in the autumn and winter months. April’s temperatures were 2.2 °C higher compared to the long-term average, and HTC was 0.03 (very dry). In May, HTC was 0.92 (optimal humidity). The temperature in June was 4.4 °C higher compared to the long-term average, and HTC was 0.80 (insufficient humidity). July was cool with an HTC of 1.12 (optimal humidity). In August, the HTC was 1.21 (optimal humidity).

The Lithuanian climate zone is at humid continental climate (Köppen–Geiger code: Dfb) [57]. Dfb: Warm summer, temperate climate. Wet, rainfall is evenly distributed throughout the year. The average temperature does not exceed 22 °C in any month. Frost is usually absent for 3–5 months per year. In summer, heat (30 °C and above) rarely lasts longer than a week. This subtype of the climate is widespread throughout the territories of Lithuania and the rest of the Baltic States, only, in this area, the seasonal variation of temperatures is mitigated by the effects of the sea.

### 4.3. Analytical Methods

The agrochemical properties of the soil were determined before the installation of the experiment and after harvesting. Soil samples were collected in 15 plots, about 300 g from the 0–25 cm ploughing layer of each experimental plot using a soil auger. Soil pH was determined potentiometrically in 1 n KCl extract, soil total nitrogen (N) was determined by the Kjeldahl method, available phosphorus P_2_O_5_ (P) and available potassium K_2_O (K) (mg kg^−1^ soil) were calculated using the Egner–Rim–Domingo (A–L) method, and organic carbon (%) (OC) was determined by incineration of samples at 900 °C using a Heraeus incinerator.

The plant root dry biomass was determined by the method of small monoliths (10 × 10 × 10 cm) [58] after the main crop harvest in the first year of caraway vegetative season and after the caraway harvest in the second and third years of caraway vegetative season. Samples were taken from two soil layers: 0–10 cm and 10–20 cm. The roots were washed through sieves and dried in a drying oven at 105 °C. Plant root mass was calculated in absolute dry matter content t ha^−1^.

The activity of soil hydrolases (urease and saccharase) was determined: urease—according to the method of Hofmann and Schmidt (1953); saccharase—according to the method of Hofmann and Seegerer (1950), modified by A. I. Chunderova (1973) [59] after the main crop harvest in the first year of caraway vegetative season and after the caraway harvest in the second and third year of caraway vegetative season. Soil samples were taken from 15 plots in each field with a soil drill at a depth of 0 to 25 cm. Samples of natural moisture were dried in boxes at laboratory temperature.

### 4.4. Statistical Analysis

The research data was statistically evaluated by one-way analysis of variance (ANOVA) using ANOVA software from the statistical analysis package SELEKCIJA [60]. The Duncan criterion was used to assess the significance of the differences. Differences between the averages of treatments marked with different letters are significant at the 95% confidence level (*p* < 0.05). There are no significant differences when *p* > 0.05.

Principal component analysis (PCA) was used to determine the correlation between different parameters in the multi-cropping system. PCA creates new artificial variables (principal components) based on the variables (features) that were analysed. Its main assumption is the possibility of visualizing the relationships of individual variables on a two-dimensional graph showing the coordinate system of the first two principal components. Based on the position of the vectors in space, it can be determined which features are correlated with each other. The smaller the angle between the vectors, the stronger the positive correlation. When the vectors are aligned on the same line but in opposite directions, there is a strong negative correlation between the variables. However, when the vectors are at an angle close to 90 degrees, no correlation occurs. Statistical analysis was performed using Statistica 10 software package (TIBCO Software Inc., Palo Alto, CA, USA).

## 5. Conclusions

The soil of binary and trinary crops had a better nutrient balance compared to the soil of sole crops. The total nitrogen, organic carbon, available phosphorus and potassium in the soil increased the most in binary and trinary crops during the third year of caraway cultivation compared to the initial level.

In the second and third years of caraway cultivation, significantly higher levels of total nitrogen and available phosphorus were found in the soil of multi-cropping system compared to sole crop. The soil organic carbon content increased significantly—from 3.8 to 17.8%—in the multi-cropping system in 2017 to 2019. In the third year of caraway cultivation, the available potassium content was found to be significantly higher in the soil of binary and trinary crops compared to the soil of sole crops.

In the first, second, and third years of caraway cultivation, plant root biomass in binary and trinary crops was significantly higher than that in sole crops.

The highest levels of saccharase and urease enzymes were found in trinary crop in the third year of caraway cultivation. The levels of saccharase and urease enzymes were most strongly correlated with the levels of total nitrogen, organic carbon, and available potassium in the soil.

The results of these studies suggest that multi-cropping is important for soil conservation and sustainable agro-ecosystems.

## Figures and Tables

**Table 1:** Total nitrogen content in the soil of sole, binary, and trinary crops in 2017–2019.

Multi-Cropping System Crops	Total Nitrogen, mg kg^−1^		
	First Year of Caraway Vegetative Season 2017	Second Year of Caraway Vegetative Season 2018	Third Year of Caraway Vegetative Season 2019
Sole			
Spring barley (SB-SB-SB)	0.114 ± 0.005 bcd	0.116 ± 0.012 b	0.108 ± 0.004 c
Spring wheat (SW-SB-SB)	0.111 ± 0.008 cd	0.115 ± 0.009 b	0.110 ± 0.005 c
Pea (P-SB-SB)	0.109 ± 0.003 d	0.131 ± 0.004 a	0.128 ± 0.004 ab
Caraway (CA-BF)	0.123 ± 0.005 abcd	0.117 ± 0.003 b	0.109 ± 0.003 c
Binary			
S. barley + Caraway (SB-CA)	0.132 ± 0.006 a	0.132 ± 0.002 a	0.129 ± 0.008 a
S. wheat + Caraway (SW-CA)	0.124 ± 0.011 abc	0.127 ± 0.011 ab	0.126 ± 0.006 ab
Pea + Caraway (P-CA)	0.128 ± 0.010 ab	0.130 ± 0.018 a	0.120 ± 0.005 b
Trinary			
S. barley + Caraway + W. clover (SB-CA-WC)	0.123 ± 0.012 abcd	0.130 ± 0.012 a	0.131 ± 0.005 a
S. wheat + Caraway + W. clover (SW-CA-WC)	0.122 ± 0.013 abcd	0.130 ± 0.010 a	0.130 ± 0.006 a
Pea + Caraway + W. clover (P-CA-WC)	0.130 ± 0.009 a	0.133 ± 0.011 a	0.132 ± 0.002 a

Note. Differences between the averages of treatments marked with different letters (a, b, c, d) are significant (*p* < 0.05). Mean ± standard deviation.

**Table 2:** Organic carbon content in the soil of sole, binary, and trinary crops in 2017–2019.

Multi-Cropping System Crops	Organic Carbon, mg kg^−1^		
	First Year of Caraway Vegetative Season 2017	Second Year of Caraway Vegetative Season 2018	Third Year of Caraway Vegetative Season 2019
Sole			
Spring barley (SB-SB-SB)	0.92 ± 0.03 cd	1.00 ± 0.05 bc	1.40 ± 0.09 b
Spring wheat (SW-SB-SB)	0.90 ± 0.06 d	0.98 ± 0.04 c	1.34 ± 0.07 b
Pea (P-SB-SB)	0.89 ± 0.03 d	1.06 ± 0.14 abc	1.54 ± 0.07 a
Caraway (CA-BF)	1.05 ± 0.02 a	1.03 ± 0.02 abc	1.30 ± 0.04 b
Binary			
S. barley + Caraway (SB-CA)	1.07 ± 0.04 a	1.16 ± 0.04 a	1.58 ± 0.04 a
S. wheat + Caraway (SW-CA)	1.05 ± 0.02 a	1.13 ± 0.08 a	1.56 ± 0.12 a
Pea + Caraway (P-CA)	0.97 ± 0.05 bc	1.10 ± 0.06 ab	1.54 ± 0.03 a
Trinary			
S. barley + Caraway + W. clover (SB-CA-WC)	0.96 ± 0.04 bc	1.06 ± 0.03 abc	1.59 ± 0.11 a
S. wheat + Caraway + W. clover (SW-CA-WC)	1.06 ± 0.05 a	1.11 ± 0.08 ab	1.55 ± 0.11 a
Pea + Caraway + W. clover (P-CA-WC)	1.02 ± 0.04 ab	1.10 ± 0.03 b	1.60 ± 0.06 a

Note. Differences between the averages of treatments marked with different letters (a, b, c, d) are significant (*p* < 0.05). Mean ± standard deviation.

**Table 3:** Available phosphorus content in the soil of sole, binary, and trinary crops in 2017–2019.

Multi-Cropping System Crops	Available Phosphorus, mg kg^−1^		
	First Year of Caraway Vegetative Season 2017	Second Year of Caraway Vegetative Season 2018	Third Year of Caraway Vegetative Season 2019
Sole			
Spring barley (SB-SB-SB)	229 ± 37.5 e	193 ± 17.6 c	254 ± 17.9 d
Spring wheat (SW-SB-SB)	257 ± 6.08 c	221 ± 26.1 bc	226 ± 28.6 e
Pea (P-SB-SB)	229 ± 33.4 e	254 ± 25.3 abc	252 ± 27.9 de
Caraway (CA-BF)	260 ± 26.7 cd	251 ± 44.0 abc	258 ± 43.8 de
Binary			
S. barley + Caraway (SB-CA)	309 ± 14.8 a	311 ± 16.3 a	338 ± 41.0 a
S. wheat + Caraway (SW-CA)	278 ± 18.5 bc	282 ± 26.5 ab	294 ± 28.3 bc
Pea + Caraway (P-CA)	235 ± 27.0 de	247 ± 29.3 abc	260 ± 14.3 cd
Trinary			
S. barley + Caraway + W. clover (SB-CA-WC)	291 ± 12.2 ab	279 ± 23.9 ab	304 ± 26.4 b
S. wheat + Caraway + W. clover (SW-CA-WC)	282 ± 13.9 bc	269 ± 19.1 ab	282 ± 36.0 bc
Pea + Caraway + W. clover (P-CA-WC)	230 ± 27.1 e	222 ± 28.5 abc	244 ± 33.0 e

Note. Differences between the averages of treatments marked with different letters (a, b, c, d, e) are significant (*p* < 0.05). Mean ± standard deviation.

**Table 4:** Available potassium content in the soil of sole, binary, and trinary crops in 2017–2019.

Multi-Cropping System Crops	Available Potassium, mg kg^−1^		
	First Year of Caraway Vegetative Season 2017	Second Year of Caraway Vegetative Season 2018	Third Year of Caraway Vegetative Season 2019
Sole			
Spring barley (SB-SB-SB)	121 ± 16.3 bc	124 ± 8.83 a	145 ± 4.24 c
Spring wheat (SW-SB-SB)	120 ± 10.0 bc	119 ± 23.3 a	140 ± 18.1 c
Pea (P-SB-SB)	120 ± 20.9 bc	130 ± 5.56 a	149 ± 2.87 bc
Caraway (CA-BF)	126 ± 5.32 abc	118 ± 26.1 a	143 ± 24.5 a
Binary			
S. barley + Caraway (SB-CA)	135 ± 5.12 ab	132 ± 17.7 a	212 ± 24.6 a
S. wheat + Caraway (SW-CA)	132 ± 12.6 abc	125 ± 13.8 a	186 ± 25.8 a
Pea + Caraway (P-CA)	120 ± 7.12 bc	120 ± 18.1 a	194 ± 19.8 a
Trinary			
S. barley + Caraway + W. clover (SB-CA-WC)	139 ± 2.75 a	135 ± 26.6 a	236 ± 39.2 a
S. wheat + Caraway + W. clover (SW-CA-WC)	118 ± 16.9 c	126 ± 34.9 a	197 ± 54.9 a
Pea + Caraway + W. clover (P-CA-WC)	117 ± 9.78 c	131 ± 8.62 a	203 ± 45.0 a

Note. Differences between the averages of treatments marked with different letters (a, b, c) are significant (*p* < 0.05). Mean ± standard deviation.

**Table 5:** Scheme for the use of pesticides in the experiment.

Name of Pesticide		Type		Active Substance				Amount	Abbreviation	
Fenix		herbicide		aclonifen 600 g L^−1^				3.00 L ha^−1^	F	
Signum		fungicide		boscalid 267 g kg^−1^ + pyraclostrobin 67 g kg^−1^				0.50 L ha^−1^	S	
Cyperkill 500 EC		insecticide		cypermethrin 500 g L^−1^				0.05 L ha^−1^	C	
Elegant 2 FD		herbicide		florasulam 6.25 g L^−1^ + 2.4-D 300 g L^−1^				0.40 L ha^−1^	E	
Karate Zeon 5 CS		insecticide		lambda-cyhalothrin 50 g L^−1^				0.14 L ha^−1^	KZ	
Bumper 25 EC		fungicide		propiconazole 250 g L^−1^				0.50 L ha^−1^	B	
Bulldock 025 EC		insecticide		beta-cyfluthrin 25 g L^−1^				0.30 L ha^−1^	Bu	
Miradol 250 SC		fungicide		azoxystrobin 250 g L^−1^				0.60 L ha^−1^	M	
Trimmer		herbicide		tribenuron-methyl 500 g kg^−1^				0.10 kg ha^−1^	T	
		2017			2018			2019		
		T1	T2	T3	T1	T2	T3	T1	T2	T3
Sole	SB-SB-SB	–	E + KZ **	B **	–	E + KZ **	M + Bu **	–	E + T **	B **
	SW-SB-SB	–	E + KZ **	B **	–	E + KZ **	M + Bu **	–	E + T **	B **
	P-SB-SB	F *	–	S + C **	–	E + KZ **	M + Bu **	–	–	B **
	CA-BF	F *	–		–	–	–	–	–	–
Binary	SB-CA	–	–	B **	–	–	–	–	–	–
	SW-CA	–	–	B **	–	–	–	–	–	–
	P-CA	F *	–	S + C **	–	–	–	–	–	–
Trinary	SB-CA-WC	–	–	B **	–	–	–	–	–	–
	SW-CA-WC	–	–	B **	–	–	–	–	–	–
	P-CA-WC	–	–	S + C **	–	–	–	–	–	–

Note. CA—caraway; SB—spring barley; SW—spring wheat; P—pea; WC—white clover; BF—bare fallow; *—after sowing; **—growing season; T1—first spray; T2—second spray; T3—third spray.

## Data Availability

Not applicable.
